# Mitochondrial dysfunction in mandibular hypoplasia, deafness and progeroid features with concomitant lipodystrophy (MDPL) patients

**DOI:** 10.18632/aging.203910

**Published:** 2022-02-23

**Authors:** Michela Murdocca, Paola Spitalieri, Angela Cappello, Fiorella Colasuonno, Sandra Moreno, Eleonora Candi, Maria Rosaria D'Apice, Giuseppe Novelli, Federica Sangiuolo

**Affiliations:** 1 Department of Biomedicine and Prevention, Tor Vergata University, Rome 00133, Italy; 2 Department of Experimental Medicine, Tor Vergata University, Rome 00133, Italy; 3 Istituto Dermopatico dell'Immacolata IDI-IRCCS, Rome 00167, Italy; 4 Department of Science, LIME, University Roma Tre, Rome 00146, Italy; 5 IRCCS Fondazione Santa Lucia, Rome 00179, Italy

**Keywords:** premature aging syndrome, MDPL syndrome, mitochondria, SOD2, ROS production, metformin, autophagy, FIB/SEM

## Abstract

Mandibular hypoplasia, Deafness and Progeroid features with concomitant Lipodystrophy is a rare, genetic, premature aging disease named MDPL Syndrome, due to almost always a *de novo* variant in *POLD1* gene, encoding the DNA polymerase δ. In previous *in vitro* studies, we have already described several hallmarks of aging, including genetic damage, telomere shortening, cell senescence and proliferation defects. Since a clear connection has been reported between telomere shortening and mitochondria malfunction to initiate the aging process, we explored the role that mitochondrial metabolism and activity play in pathogenesis of MDPL Syndrome, an aspect that has not been addressed yet. We thus evaluated mtDNA copy number, assessing a significant decrease in mutated cells.

The expression level of genes related to mitochondrial biogenesis and activity also revealed a significant reduction, highlighting a mitochondrial dysfunction in MDPL cells. Even the expression levels of mitochondrial marker SOD2, as assessed by immunofluorescence, were reduced. The decrease in this antioxidant enzyme correlated with increased production of mitochondrial ROS in MDPL cells, compared to WT. Consistent with these data, Focused Ion Beam/Scanning Electron Microscopy (FIB/SEM) analysis revealed in MDPL cells fewer mitochondria, which also displayed morphological abnormalities. Accordingly, we detected autophagic vacuoles containing partially digested mitochondria.

Overall, our results demonstrate a dramatic impairment of mitochondrial biogenesis and activity in MDPL Syndrome. Administration of Metformin, though unable to restore mitochondrial impairment, proved efficient in rescuing nuclear abnormalities, suggesting its use to specifically ameliorate the premature aging phenotype.

## INTRODUCTION

MDPL Syndrome is a rare, genetic, premature aging disease (OMIM #615381) characterized by Mandibular hypoplasia, Deafness and Progeroid features with concomitant Lipodystrophy. It has been associated with a *de novo* variant in *POLD1* gene, encoding DNA polymerase δ.

In previous *in vitro* studies [1, 2] we demonstrated several hallmarks of senescence and of *LMNA*-linked progeria in MDPL fibroblasts. In particular, MDPL cells exhibited micronuclei, nuclear architecture abnormalities, and prelamin A aggregation. The aged phenotype is also accompanied by a decrease of cell growth, cellular aging and proliferation blockage in G0/G1 phase. Furthermore, telomere shortening was faster in MDPL cells, proposing their malfunction as a relevant trait in MDPL syndrome.

Current papers have reported that telomere shortening may also influence mitochondria activity through many pathways to begin the aging process, among which the nuclear-mitocondrial signalling [3]. Adaptive response to such damage involves peroxisome proliferator-activated receptor gamma co-activator 1a (PGC-1a), a master regulator of mitochondrial biogenesis and activity, which is activated by SIRT1. In turn, PGC-1a induces the expression of mitocondrial antioxidant enzyme superoxide dismutase 2 (SOD2).

Mitochondria are critically involved in biological aging [4, 5]. They form a complex and tubular system moving along microtubules and actin fibres [6, 7]. To sustain a functional population, mitochondria go through a fine equilibrium between fusion and fission, while damaged mitochondria are cleared via autophagy and replaced by new mitochondria [8, 9]. Fusion importantly contributes to mtDNA preservation. Deficiency in mtDNA maintenance may reveal a quantitative decrease in mtDNA copy number [10]. Mitochondria are the main intracellular source of reactive oxygen species (ROS) [11], so that dysfunctional mitochondria can cause rising amounts of ROS, consequently determining DNA and protein damage [12]. Excessive ROS production, caused by telomere shortening, can regulate the DNA damage response (DDR) and maintain persistent cellular senescence [11].

In Hutchinson-Gilford Progeroid Syndrome (HGPS) fibroblasts, as well as in HGPS mouse models a strong downregulation of mitochondrial oxidative phosphorylation proteins, reduced ATP levels, and mitochondrial impairment were described [13–14]. However, it is not clear how mitochondrial dysfunction contributes to the premature aging phenotypes linked with HGPS.

Recently, a novel MAD progeroid syndrome (MADaM: Mandibuloacral dysplasia associated to MTX2) has been described with clinical characteristics evoking HGPS. MADaM is caused by recessive mutations in *MTX2* gene, encoding for Metaxin-2 (MTX2), an outer mitochondrial membrane (OMM) protein. MTX2 loss determined mitochondrial architecture fragmentation, reduced oxidative phosphorylation, augmentation of senescence and autophagy, and decreased proliferation. Furthermore, it secondarily impacts nuclear morphology resembling HGPS and other Progeroid Laminopathies, probably emphasizing common clinical features [15].

Likewise to the majority of the rare diseases, studies on MDPL syndrome are aimed also to identify a successful therapy. Metformin is mainly an anti-diabetic agent, able to suppress glucose production and to alleviate insulin resistance. Even though the molecular mechanisms which explain its action remain largely unknown, the drug has recently been shown to enhance mitochondrial respiratory activity [16, 17]. Metformin is more beneficial than any other antidiabetic drug in reducing age-related diseases and is considered as a potential “geroprotector”, due to its ability in increasing proliferation and inhibiting senescence [16, 17]. Also a recent study addressed the potential of metformin as an anti-aging drug, using it for treating HGPS cell phenotype. Its administration allowed a decrease of progerin expression and of nuclear shape abnormalities, thus proposing a therapeutic potential for considering this drug as a good candidate for Progeroid diseases [18, 19].

The purpose of this work is to broaden the knowledge on the involvement of mitochondrial metabolism and activity in the pathogenesis of MDPL Syndrome, aspects that have not been elucidated yet. Our results indicated an altered mitochondrial phenotype in MDPL cells. Pharmacological treatment of these cells with metformin suggested that the drug is able to restore nuclear abnormalities, but none of the mitochondrial alterations.

## RESULTS

### MtDNA copy number and mitochondrial biogenesis in MDPL and WT HDFs

The copy number of mtDNA reveals the quantity of mitochondria and may shift depending to the cell energy requirements, as well as the physiological or environmental conditions. Several studies have described a decrease in mtDNA content in age-associated diseases [20–23].

**Figure 1:**
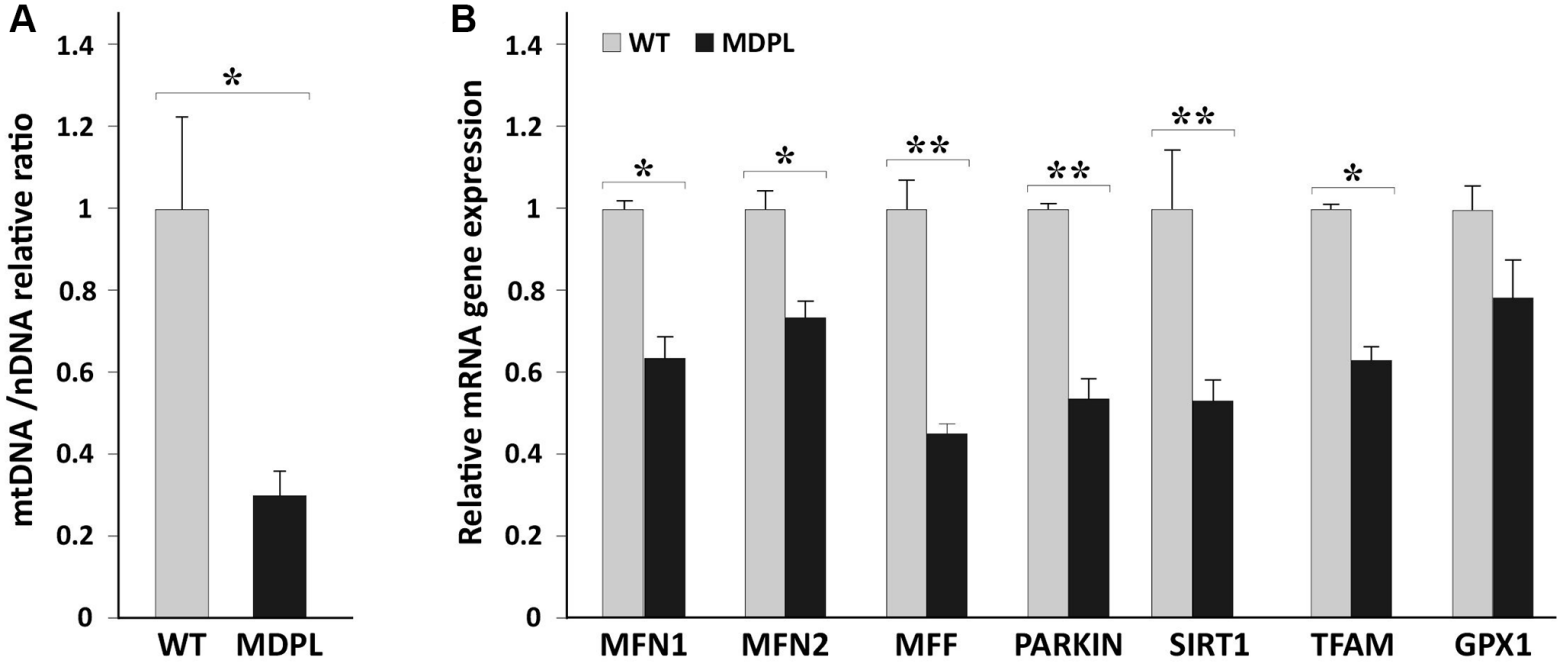
**mtDNA copy number and quantification of mitochondrial markers in HDFs WT and MDPL.** (**A**) Comparison of mtDNA copy number between WT and MDPL. mtDNA copy number are reported as mean ± standard deviation. ^*^p < 0.05. (**B**) Quantification of mRNA levels of MFN1, MFN2, MFF, PARKIN, SIRT1, TFAM transcription factors, GPX1 in MDPL and control fibroblasts (WT). Data are from three independent experiments and represented as mean ± SD; (^*^p < 0.05 ^**^p < 0.01).

In light of this, we analyzed the amount of mtDNA in MDPL (*N* = 2) and WT (*N* = 2) fibroblasts, reporting a significant decrease of mtDNA copy number in patients compared to control group (^*^p = 0.02; Figure 1A). MtDNA copy number analysis was performed as described by Rooney et al. [20].

Mitochondria are part of a comprehensive network maintained by a sophisticate balance between mitochondrial biogenesis, fission and fusion, and mitophagy [24, 25]. To evaluate these processes, we examined the expression of TFAM, determining the abundance of mitochondrial genome, *MFN1* and *MFN2*, known as fusion-related genes, *MFF*, fission related genes and *PARKIN*, which is implicated in mitophagy [26]. Remarkably, the mRNA levels of all these genes were found to be significantly decreased in MDPL fibroblasts by Real time q-PCR (Figure 1B). Also GPX1 expression resulted to be decreased together with its critical downstream target i.e., SIRT1, representing a key metabolic sensor that acts on different cellular processes, such as energy metabolism, stress response and aging [27].

Overall, these data highlight a significant mitochondrial dysfunction and an impaired metabolism in MDPL cells.

### Mitochondrial alterations in MDPL cells

**Figure 2:**
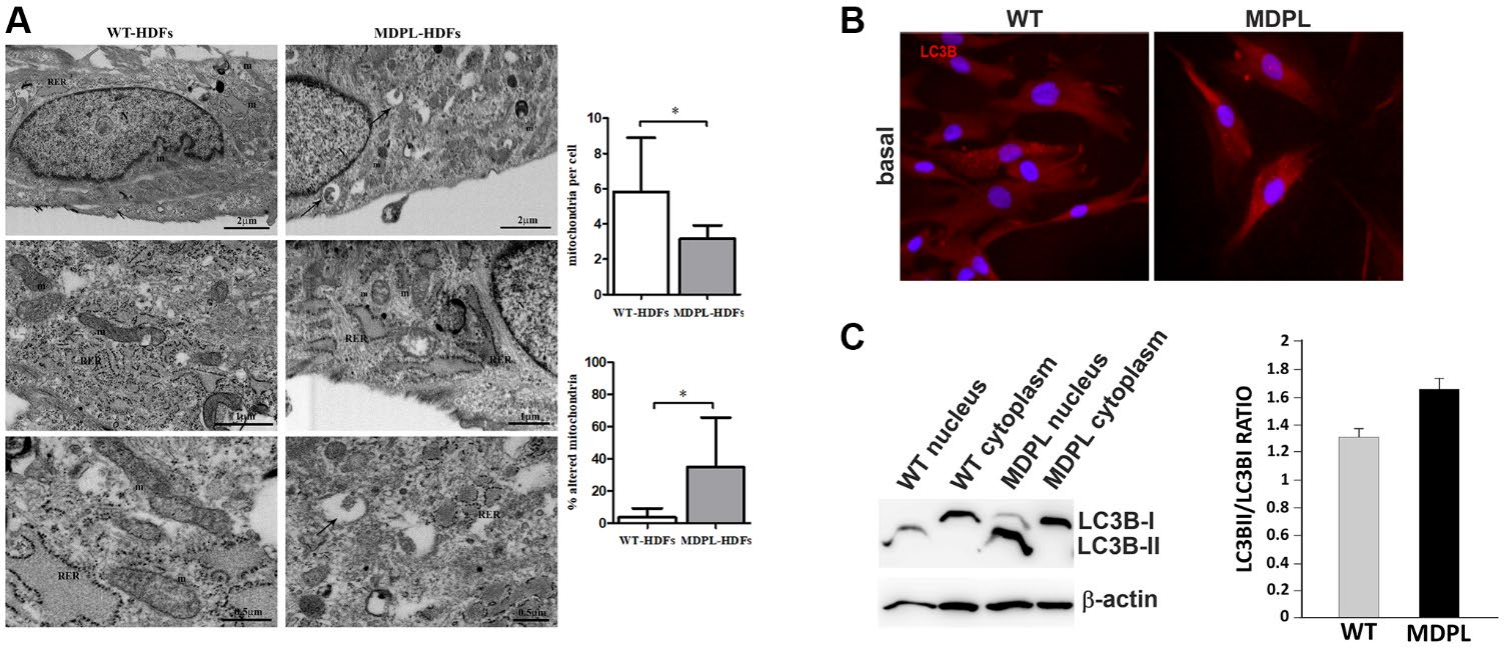
**Ultrastructural analyses of mitochondria and their autophagic activity.** (**A**) FIB/SEM analysis of MDPL-HDFs vs. WT fibroblasts. Healthy cells (on the left) show regular nucleus (N), several mitochondria (m) and abundant rough endoplasmic reticulum (RER). By contrast, MDPL-HDFs cells (on the right) display fewer RER cisternae, while smooth endoplasmic reticulum (SER) and Golgi apparatus are more prominent than in WT. Diseased cells also show several autophagosomes (black arrows), often containing partially digested mitochondria. Statistical analysis demonstrates significant decreased number of mitochondria, which are also significantly more damaged, than their normal counterpart (^*^p < 0.05, for both parameters). (**B**) Representative image of immunofluorescence analysis of LC3 in WT and MDPL HDFs. (**C**) Western blot densitometric analysis of the LC3-II/I ratio. Data are presented as means ± SD. β-actin was used as control.

Ultrastructural analysis highlighted remarkable mitochondrial alterations in MDPL fibroblasts, when compared to healthy cells (WT) (Figure 2A). These organelles appeared altered in shape with disrupted *cristae in* MDPL-HDFs vs. WT. Statistical analysis demonstrated overall fewer mitochondria in MDPL-HDFs vs. WT (*p* < 0.05), while the number of damaged mitochondria (*p* < 0.05) resulted to be significantly higher in mutated cells.

FIB/SEM micrographs occasionally revealed the presence of autophagic vesicles in MDPL-HDFs cells inside which partially digested mitochondria are recognizable (black arrows).

### Autophagic process in MDPL cells

Microtubule-associated protein light chain 3 (LC3) conversion (LC3-I to LC3-II) is considered a valid autophagic measure, and the amount of LC3-II is directly proportional with the number of autophagosomes. Its expression was clearly visible by immunofluorescence both in WT and MDPL-HDFs (Figure 2B). Western blot analysis usually detected two separate bands: one represents LC3-I, which is cytosolic, and the other LC3-II, which is conjugated with phosphatidylethanolamine (PE) and is mainly present on isolated membranes and autophagosomes (Figure 2B). Our data, obtained after a biochemical nuclear fractionation, showed that even if a slight LC3-II increase was present in the proband, probably indicating an higher number of autophagosomes, LC3-II/LC3-I ratio seemed to remain comparable among MDPL and WT cells (Figure 2C).

### Quantification of total and mitochondrial ROS levels, SOD2 expression and JC1 staining in MDPL fibroblasts

**Figure 3:**
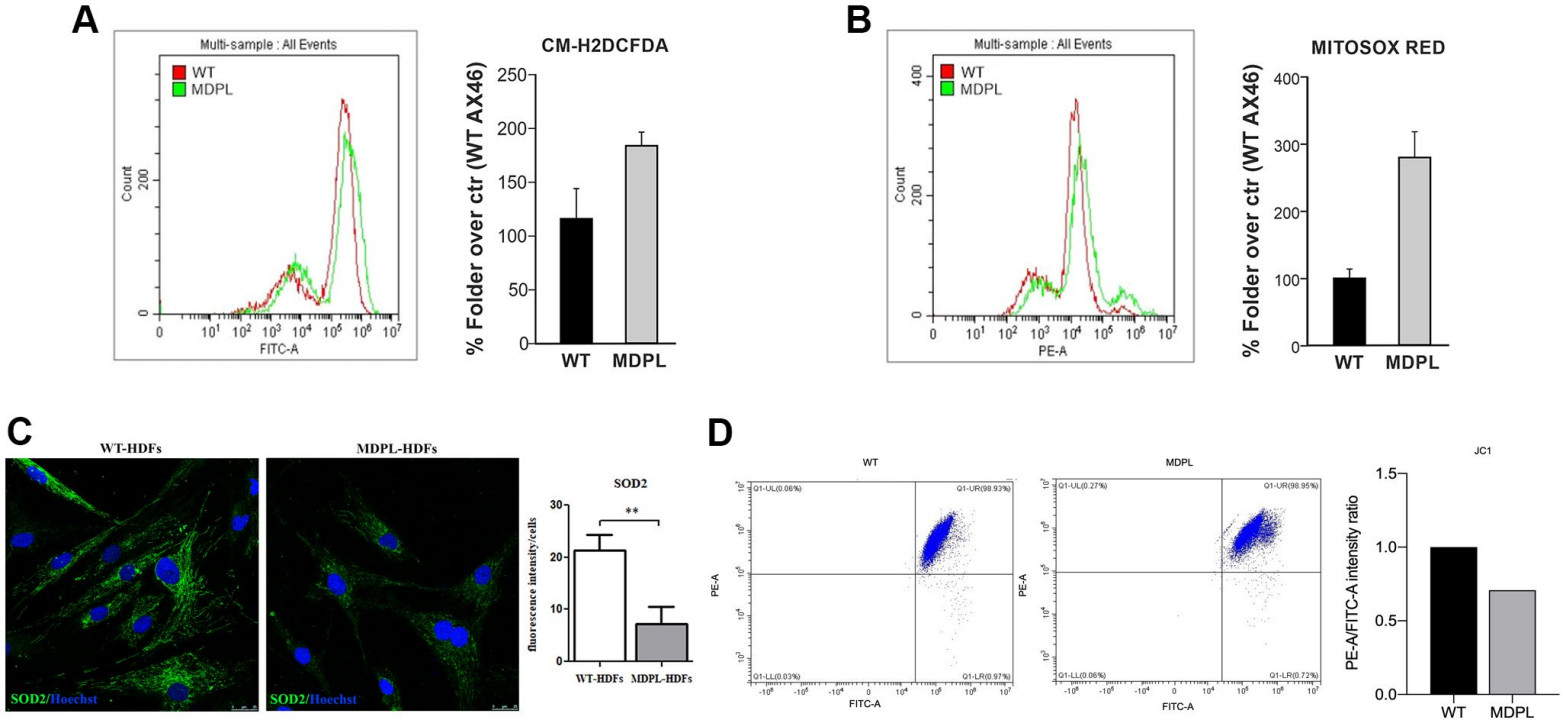
**Functional mitochondrial evaluation of MDPL and WT HDFs.** Flow cytometry quantification of (**A**) total reactive oxygen species using CM-H2DCFDA in HDFs WT and MDPL ones. (**B**) Flow cytometry quantification of mitochondrial superoxide using MitoSOX Red in HDFs WT and in MDPL ones. The error bar indicated in panels A and B is the average of two independent experiments. (**C**) Confocal analysis following SOD2 immunofluorescence and its quantification (^**^p < 0.01). (**D**) JC1 staining for flow cytometry to evaluate changes in mitochondrial potential membrane and the quantification of red/green fluorescence intensity ratio.

To determine the production of ROS in MDPL HDFs, we analyzed the quantity of total and mitochondrial superoxide using the chloromethyl derivate of 2′,7′-dichlorodihydrofluorescein diacetate (CM-H2DCFDA) and MitoSOX Red staining, respectively. CM-H2DCFDA staining showed a trend increase of the intracellular hydrogen peroxide content (~60%) in MDPL fibroblasts compared to the WTs (Figure 3A). Furthermore, MitoSOX Red staining, specific for mitochondrial O_2_^-^detection, revealed a strong increase trend (~180%) in MDPL cells (Figure 3B).

Immunofluorescence localization of SOD2 was examined. This protein represents a functional mitochondrial marker and in the same time a major ROS-scavenging enzyme. Confocal analysis (Figure 3C) revealed significantly decreased expression of the antioxidant enzyme in MDPL-HDFs compared to WT cells (*p* < 0.01).

In addition, JC1 staining for flow cytometry, indicated a tendency to decrease of the red (PE-A)/green (FITC-A) intensity ratio. This evidence showed that the mitochondrial membranes of MDPL cell lines resulted depolarized, when compared to the mitochondrial membranes of WT cell lines (Figure 3D).

### Metformin treatment: quantification of total and mitochondrial ROS

**Figure 4:**
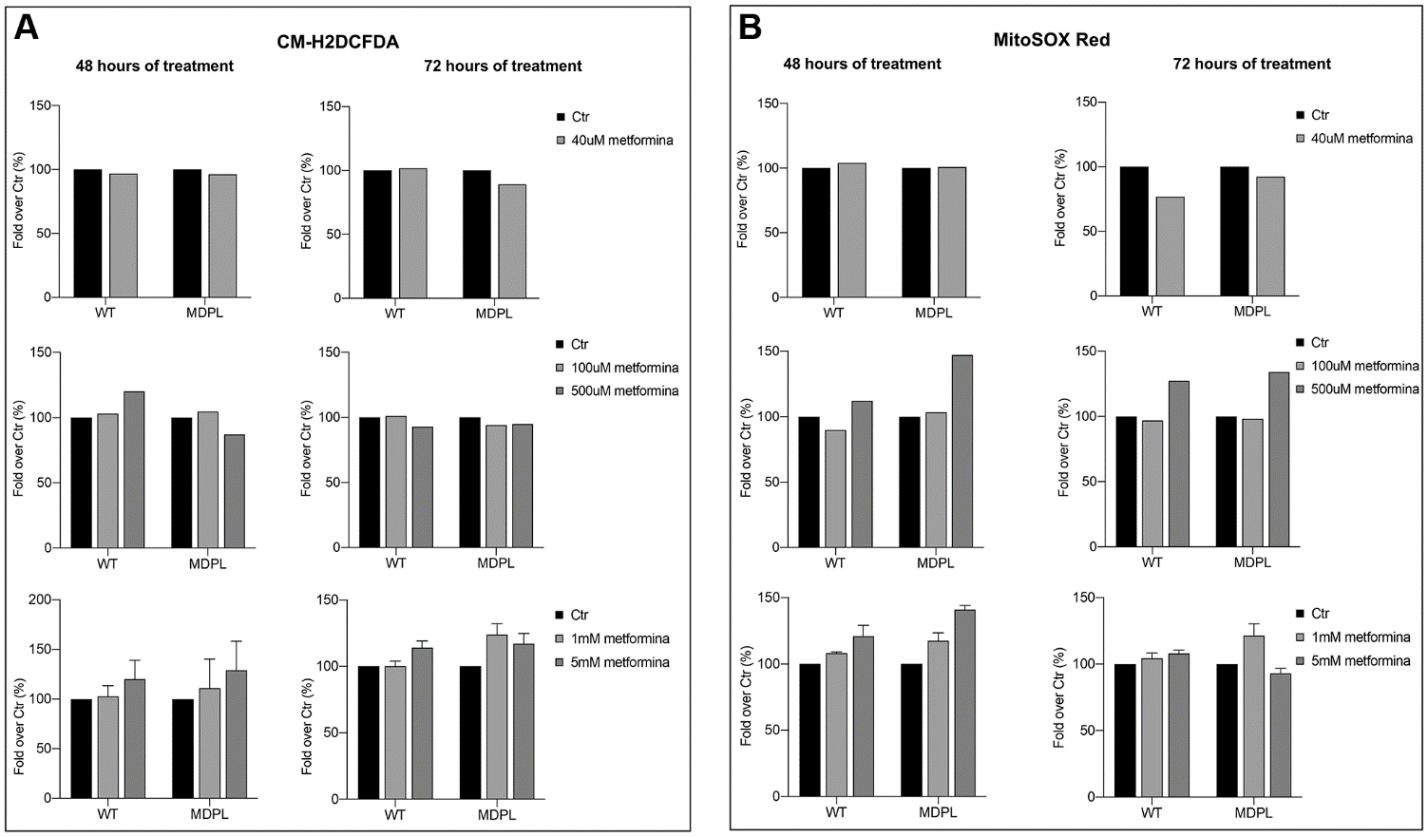
**Total and mitochondrial superoxide content after metformin treatment at different concentrations and timing.** Flow cytometry quantification of total ROS (**A**) using CM-H2DCFDA in WT and in POLD1 human dermal fibroblasts after 48 and 72 hours of 40 uM, 100 uM, 500 uM, 1 and 5 mM of metformin treatment. (**B**) Flow cytometry quantification of mitochondrial superoxide using MitoSOX Red in WT and in POLD1 human dermal fibroblasts after 48 and 72 hours of 40 uM, 100 uM, 500 uM, 1 and 5 mM of metformin treatment.

To further understand the effect of different concentrations of metformin on WT and MDPL cells in the production of intracellular hydrogen peroxide, we performed a cytofluorimetric analysis of total ROS with CM-H2DCFDA staining. After 48 hours of treatment with a low concentration of metformin (40 uM), no differences in intracellular ROS production were evidenced (Figure 4; left panel). Using higher concentrations of metformin (100 uM), the WT HDFs showed a tendential increase of ROS; at 500 uM the number of total ROS seemed to decrease in MDPL cell line. By contrast, at higher concentrations (1–5 mM) a tendential increase of total ROS production was evidenced.

Moreover, we would observe the effect of different metformin concentrations also in mitochondrial superoxide anion production. To analyze the amount of mitochondrial ROS, we stained the cells with MitoSOX red after 48 and 72 hours of metformin treatment, we then measured the red fluorescence using a cytometer. Data presented in Figure 4 showed that a low concentration of metformin determines no differences in mitochondrial ROS after 48 hours of treatment while a tendency to decreasing of mitochondrial ROS was observed at 72 h in both cells (Figure 4; right panel). No significant effect is shown using 100 uM of metformin at both 48 and 72 h, although a strong increase of the MitoSOX fluorescence is observable at higher doses (500 uM and 1–5 mM), but at 5 mM 72 h fluorescence is decreased in MDPL.

### Metformin treatment: nuclear envelope characterization and micronuclei assessment

**Figure 5:**
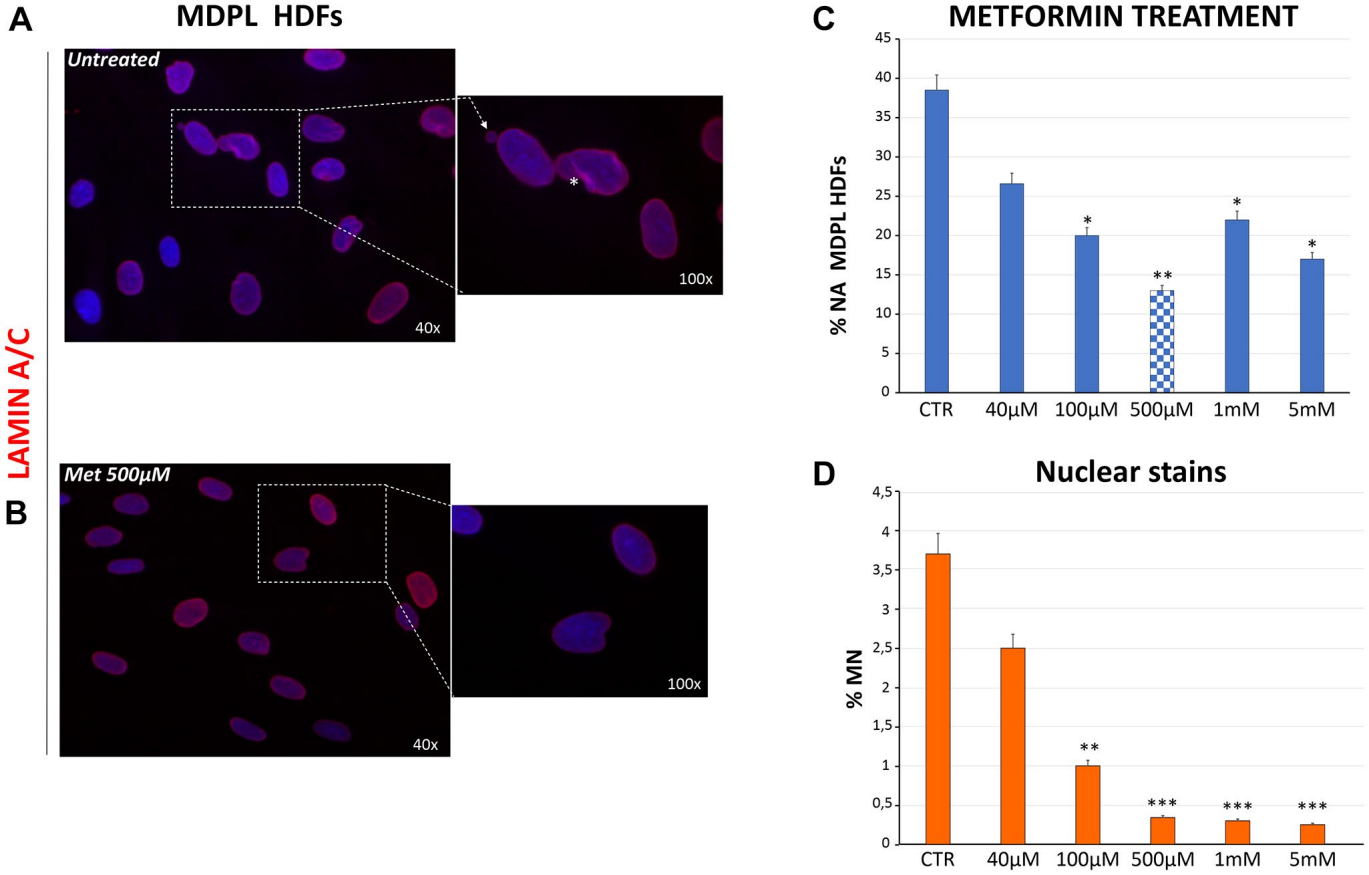
**Evaluation of nuclear shape organization after metformin treatment at different concentrations.** (**A**) Representative image of nuclear shape organization observed in MDPL HDFs stained for lamin A/C (red), showing the presence of membrane invaginations (asterisks), and micronuclei (white arrows). (**B**) Representative image of lamin A/C immunostaining in MDPL HDFs following 48 h of treatment with 500 μM of metformin, in which a clear reduction in nuclear anomalies is evident. (**C**) Evaluation of aberrant nuclear alteration (% NA) in MDPL HDFs treated for 48 h with increasing doses of metformin. Each value represents the mean ± SD. of the analysis of 300 cells observed in three independent experiments (^*^p < 0.05; ^**^p < 0.01). Values are displayed as the average percentages of two different patients (**D**) Percentage of micronuclei (MN) encountered in MDPL-HDFs after 48 h of metformin treatment. The data have been obtained counting the micronuclei after Hoechst 33342 nuclear staining for fluorescence imaging. Each value represents the mean ± S.D. of the analysis of 300 cells for three independent experiments (^**^p-value <0.01; ^***^p-value <0.001). Values are displayed as the average percentages of two different patients. Hoechst 33342 nuclear staining (blue). Magnification 40× and 100×. Abbreviations: HDFs: human dermal fibroblasts; Met: metformin; MN: micronuclei; NA: aberrant nuclear alteration; Untreat: untreated cells.

We previously described different alterations of nuclear shape in MDPL HDFs, together with a high frequency of micronuclei [1, 2]. In order to analyze the therapeutic potential of metformin for the MDPL disease, HDFs were treated with metformin administration to explore any effects on both impairment of nuclear shape and presence of micronuclei. Nuclear shape disorganization in MDPL HDFs was revealed using mature lamin A/C staining (Figure 5A). At the same number of passages, primary MDPL fibroblasts present approximately 40% of abnormal nuclei, as compared with WT HDFs, which reveal less than 5% of nuclear abnormalities as part of the physiological ageing process (data not shown). After metformin treatment, we have observed in MDPL cells an ameliorative effect already at a concentration 40 μM (40% untreated vs. 26.6% treated) increasing at 100 μM (40% untreated vs. 20% treated) and even more at 500 μM (40% untreated vs. 13% treated) (Figure 5B and 5C). Therefore, the aberrant nuclear alterations showed show an improvement trend in a statistically significant manner as drug concentration increases. Anyway, at 1 and 5 mM there is a trend of reduction of nuclear alteration respect to untreated cells.

The curative potential of metformin for MDPL was then studied by measuring its effect on micronuclei presence. Importantly, an evident dose-dependent decrease of micronuclei was also revealed reaching 0.3% in MDPL cells treated with 500 uM concentrations, the most efficacious also for nuclear abnormalities (Figure 5D). As expected, treatment with metformin had no effect in WT cells (data not shown).

## DISCUSSION

Premature-ageing syndromes are a group of rare genetic diseases, evoking characteristics of accelerated ageing. They usually resulted from mutations in genes encoding for proteins needed for nuclear lamina network, preservation of genome stability and DNA repair, mitochondrial activity and other cellular processes.

Mandibular hypoplasia, Deafness, Progeroid features and Lipodystrophy Syndrome (MDPL; OMIM #615381) belongs to this group. MDPL is an extremely rare (prevalence of <1/1,000,000) progeroid syndrome with characteristic of generalized lipodystrophy, dysmorphic features, telangiectasia, early onset hearing loss, insulin resistance and dyslipidemia.

Less than thirty patients have so far been studied as having POLD1-related MDPL Syndrome [28], and most of them carry the recurrent POLD1 p.Ser605del *de novo* mutation. *POLD1* gene encodes human polymerase delta (Polδ)1, conferring proofreading activities of DNA polymerase [29], which in turn is involved in DNA replication and in multiple DNA repair mechanisms [30–33].

In a previous paper [2], we elucidated some aspects related to p.Ser605del mutation in MDPL fibroblasts and identified nuclear and cellular alterations also encountered in other Progeroid Syndromes. These include an impairment of nuclear envelope, accumulation of prelamin A, altered cell growth, cellular senescence, compromised ability to repair DNA double-strand breaks, presence of micronuclei and an increase rate of telomere shortening.

In the context of organelle response to nuclear DNA damage, mitochondria have recently attracted considerable attention, but their role in MDPL Syndrome remained obscure. Mitochondria play a central role in cellular processes contributing to aging and form a complex network of tubulare structures, undergoing fusion and fission events, to meet changing cell energy requirements.

In the present work, we have studied mitochondrial ultrastructural features, number and functionality in primary cultures of fibroblasts derived from two MDPL patients. Damaged mitochondria were significantly more numerous in diseased than in healthy cells. These organelles showed overall altered shape and profound inner membrane abnormalities, as *cristae* were hardly recognisable. Besides, overall reduced number of mitochondria in MDPL cells are also demonstrated by FIB/SEM analysis. This result is consistent with immunofluorescence data showing significantly lower levels of the mitochondrial marker SOD2 [34]. All these data are consistent with reduced expression of genes involved in mitochondrial biogenesis, turnover and metabolism, as seen in other cellular models of genetic diseases [35–37].

Mitochondrial DNA copy number, a measure of mitochondrial genome quantity is usually used as a marker of the mitochondria’s response to oxidative stress, as well as general dysfunction. Different works have demonstrated that mtDNA decreases with age [22, 23] and is positively associated with telomere length. The mitochondrial DNA copy number in MDPL cells is strongly compromised and closely connected with the down modulation of *TFAM* transcript, which in turn regulates mtDNA maintenance, function and content.

Characteristics as a decrease in mtDNA copy number, the alteration in nucleoid size and an associated clustering are usually linked with disorganized *cristae* or the other way round. Also, alteration of mitochondrial dynamics has distinct impacts on *cristae* and mtDNA integrity. At least we can say that loss of mitochondrial dynamics frequently drives to abnormal *cristae* development and that aberrations in *cristae* morphology frequently result in loss of mtDNA.

Thus, this altered condition in terms of integrity and quality probably leads to an impaired cellular energy metabolism. Cellular aging causes mitochondrial ROS production and their increase has been already described in HGPS cells [38, 39]. As the majority of ROS is produced inside the mitochondria, it was initially thought that ROS overproduction was the causal link between mitochondrial dysfunction and cellular aging [40–43]. On the other hand, ROS excess may even result from impaired antioxidant defences. Indeed, our immunofluorescence data showed that considerably lower levels of SOD2 protein are expressed in MDPL fibroblasts, if related to WT ones. This may make the overall picture more complex, since SOD2 is also a marker for mitochondrial biogenesis. Thus, it is conceivable that other energy metabolism related functions, including lipid metabolism, are affected.

Autophagy is important in the clearance of impaired cellular organelles including mitochondria. During autophagy, the cytoplasmic LC3-I protein is changed to LC3-II and recruited on the autophagosome membrane. Thus, we investigated LC3 expression as an autophagic marker, as it is clearly correlated with the number of autophagosomes and acts as a good indicator of autophagosome formation. At basal level a slightly increase of LC3-II is detected by Western blot analysis in MDPL cells when related to healthy ones, even if not statistically significant. Ultrastructural analysis also evidenced an abnormally prominent smooth endoplasmic reticulum and Golgi apparatus in MDPL cells, in basal conditions. Several autophagosomes, often containing partially digested mitochondria, were also detected, suggesting their selective removal by mitophagy.

Metformin is known as a drug able to combat age-related disorders [44] and improve health span. It has been in clinical use for over 60 years, demonstrating a high safety profile. Metformin is a complex drug with multiple sites of action and multiple molecular mechanisms. In spite of its widespread use, the mechanisms by which metformin exerts favorable effects on aging remain largely unknown. Experimental data suggested that the drug acts by delaying stem-cell aging, modulating mitochondrial function and lowering senescence [45–48]. It is well known that genomic instability is a pivotal hallmark of biological aging [49]. It is caused by mitochondrial ROS, DNA replication errors, chromosomal rearrangements and environmental or iatrogenic agents [50, 51]. Metformin exerted its genome protective effects by increasing DNA-damage like response and DNA repair [52]. Metformin also improves autophagy and mitochondrial function in parallel to decrease inflammaging. Mitochondria are a primary target of metformin [53–58]. Thanks to these properties, metformin was already used *in vitro* in HGPS cells to cure premature aging phenotype; its administration caused a decrease of the progerin expression reducing at the same time the abnormalities in nuclear shape architecture and reactive oxygen species formation. These results suggest a therapeutic potential for a repurposing of this drug [18, 19]. Another study reported the beneficial effects of metformin in Myotonic Dystrophy type 1 (DM1) cells evidencing a restored metabolism and mitochondrial function. DM1 resembles the appearance of a multisystem accelerated aging process, therefore revealing the efficiency of metformin treatment in a pre-clinical setting [59].

To investigate if metformin can result effective in MDPL cells, we administered different concentrations of drug *in vitro* and tested potential reversing of excessive ROS production and nuclear abnormalities, two aging traits typical of MDPL phenotype [1, 2]. While mitochondrial ROS levels appeared to be higher in MDPL cells respect to WT ones at basal level, no significantly different activity has been evidenced after metformin administration.

A significant recovery has been instead observed for both nuclear abnormalities and micronuclei number after drug treatment, reporting a clear effect at 500 uM concentration. The occurrence of micronuclei in mammalian cells has been linked to a number of mutagenic stresses. During mitosis, micronuclei originated from lagging chromatids or chromatin bridges between anaphase chromosomes and were stable for up to one cell cycle. Gene entrapment/trapped in micronuclei has the potential to cause significant phenotypic changes in cells, as well as apoptosis. The micronucleation has important implications in the genomic plasticity [60]. Several other nuclear abnormalities are induced by genotoxic stress, like buds (or blebs) [60, 61], formed from chromatin bridges and that could change into micronuclei in the course of interphase [62]. Overall, micronuclei and nuclear buds are major indexes for genome instability [2]. Nuclear envelope alterations induce genome instability and a constitutive DDR activation [63, 64]. Chronic genomic instability causes cellular effects, including chromatin structural problems and changes in cell fate. This situation induced cellular senescence, reduced regenerative capacity, altered metabolism, modified mitochondrial homeostasis, and the activation of systemic stress and proinflammatory responses. In fact, cellular senescence is a mechanism of response to chronic DNA damage described in a variety of Progeroid Syndromes, as well as natural aging [63].

Given our finding that different nuclear defects in MDPL cells are responsive to metformin treatment, it will be peculiar to determine if other cellular characteristics of aging respond to the same treatment and if organismal aging can be influenced by interference with lamin A.

Addressing the questions of why and how metformin acts is crucial to understand how the nucleus is rebuilt at the end of mitosis cycle.

To our knowledge, this is the first study addressing mitochondrial involvement in MDPL cells, by characterising these organelles both ultra-structurally and functionally. Our data strongly support a crucial and multifaceted role of mitochondrial alteration in developing premature senescence phenotype. This study also proposes metformin as a drug not to reverse damage mitochondria or function but to recover the premature aging phenotype in MDPL nuclei, though unrelated to ROS production. Further studies of the different areas connecting the cytoskeleton to the nucleoskeleton should reveal possible “mechanical” links among dysfunctional mitochondria and nuclei in patients’ cells and how reciprocally and secondarily influencing each other.

Future investigations are also needed to dissect the molecular mechanisms underlying metformin beneficial effects on nuclear damage.

## MATERIALS AND METHODS

### Mitochondrial DNA (mtDNA) copy number

DNA was extracted from MDPL and WT cells with “Wizard genomic DNA purification kit” using standard procedures. DNA quality and concentration were evaluated by NanoDrop ND-1000 Spectrophotometer (Euro-Clone). Primers amplifying a nuclear DNA region (hemoglobin subunit beta [HGB]) and a mtDNA region (NADH dehydrogenase, subunit 1, ND1).The mitochondrial copy number in fibroblasts was calculated by the equation (2 · 2(Ct (HGB)-Ct(ND1)) [20].

### RT-qPCR analyses

To extract total RNAs from cells, TRIzol Reagent (Invitrogen; Life Technologies Corporation, Carlsbad, CA, USA) was employed following the manufacturer’s instructions, and then the RNAs were treated with DNase I (RNase-free Ambion, Life Technologies Corporation, Foster City, CA, USA). One μg of RNA was reverse transcribed with the High-Capacity cDNA Archive kit (Life Technologies Corporation, Foster City, CA, USA) and used in real-time reverse transcription (RT)–polymerase chain reaction (PCR). SYBR Green chemistry (Life Technologies Corporation) and specific primers for mitochondrial and glyceraldehyde-3-phosphate dehydrogenase (GAPDH) genes were used; mRNAs were analyzed by SYBR Green (Life Technologies Corporation, Foster City, CA, USA). Quantitative measurements were ascertained using the ΔΔCt method. Primer sequences will be given upon request.

### Fractionation of fibroblast nuclei and Western blot

Cells at 70% of confluence were harvested. For nuclei to cytoplasm separation, we followed the manual of NE-PER Nuclear and Cytoplasmic Extraction Reagents (#78835; Thermo Scientific). After removing the cytoplasm supernatant, the pellets containing nuclei were resuspended in lysis buffer, as already described in Murdocca et al. [2]. After centrifugation at 16000 g of the whole nuclei lysate, the soluble fraction and the insoluble fraction of the nuclei were prepared for Western blot assay by adding Laemmli sample buffer (Bio-Rad) [2]. After saturation in 5% milk/PBS, the nitrocellulose membrane was labelled with beta-actin and anti-LC3B (NB100-2220-Novusbio). Peroxidase-conjugated secondary antibodies were used (1:10000; EMD Millipore Corporation, Billerica, MA, USA). Signals were scanned and quantified on ImageQuant LAS 4000 system.

### Ultrastructural analysis by focused ion beam/scanning electron microscopy (FIB/SEM)

For ultrastructural analysis, cells were plated in a Chamber Slide™ system and fixed in 0.5% glutaraldehyde and 2% paraformaldehyde, 0.1 M cacodylate buffer, pH 7.4 for 1 h, then post fixed in 1% osmium tetroxide in the same buffer for 45 min, in the dark. Samples were washed for 30 min and contrasted *en bloc* with 1% uranyl acetate in the dark, then gradually dehydrated in ethanol. All the steps of the above procedure were performed at 4°C. Cells were infiltrated with a mixture of ethanol and Epoxy Embedding Medium (Sigma-Aldrich™, Cat# 45359-1EA-F), then embedded in the same resin, allowing specimens to polymerize at 60°C, for 3 days. Resin-embedded cells were mounted on stubs using a self-adhesive carbon disk and gold sputtered by an Emithech K550. Regions of interest were cross-sectioned by the focused gallium ion beam of the Dualbeam FIB/SEM (Helios Nanolab, FEI, Hillsboro, OR, USA). Images of cross sections were acquired at a working distance of 2 mm using backscattered electrons and a through-the-lens detector in immersion mode with an operating voltage of 2 kV and an applied current of 0.17 Na. Images were composed in an Adobe Photoshop CS6 format.

### Metformin treatment

Skin fibroblasts were obtained and cultured from MDPL patients (MDPL HDFs) and healthy donors (WT HDFs) as already described [2], in according to The Committees on Health Research Ethics of Tor Vergata Hospital (2932/2017) and EU ethical rules.

Twenty-four hours after seeding, at p14, the HDFs were treated with different concentrations of metformin (1,1-dimethylbiguanide hydrochloride, Sigma). Cells were analyzed after 48 h and 72 h of treatment.

### Total and mitochondrial ROS production

MDPL and WT HDFs were trypsinized and stained with DMSO and CM-H2DCFDA (10 μM; Invitrogen), MitoSOX Red (5 μM; Invitrogen) and JC1 (2 μM; Invitrogen). The cells were incubated for 20 min at 37°C to allow the permeabilization of the probes and were analyzed by flow cytometry (Beckman Coulter, Cytoflex) acquiring 12000 events per sample. In detail, the CM-H2DCFDA fluorescent signal was collected in the FITC channel, MitoSOX Red in the PE-A channel and for JC1 the signal was collected in both FITC and PE-A channel.

### Immunofluorescence staining

Cells grown on coverslips have been fixed in 100% methanol at −20°C for 7 min and incubated with primary antibodies Lamin A/C (N-18; 1:100, Santa Cruz Biotechnology, INC) and LC3B (NB100-2220 Novusbio). Immunofluorescence analysis for superoxide dismutase 2 (SOD2)(1:200 anti-SOD2, ab13533, Abcam) was performed on cells fixed in 4% paraformaldehyde, and successively incubated with primary antibody and specific Alexa Fluor 568 and 488-labeled secondary antibodies (Invitrogen, Carlsbad, CA, USA) in the presence of Hoechst 33342 (sigma Aldrich). Slices were analysed under a fluorescence microscopy and images are acquired using a Zeiss (Zeiss, Thornwood, NY, USA) Axioplan 2 microscope and Leica TCS SP5 confocal microscope (Leica, Wetzlar, Germany). Representative images, captured by a Leica Application Suite software, were composed in an Adobe Photoshop CS6 format (Adobe Systems Inc., San Jose, CA, USA). ImageJ (NIH) software was used to quantify immunofluorescence intensity.

### Statistical analyses

Statistical analysis for RT-qPCR was carried out using Prism software and the SPSS program, version 25 (IBM Corp, Armonk, NY, USA). For mitochondrial morphometric analyses, 10 cells/sample were analyzed by manually counting regular and altered mitochondria. Two-way ANOVA was used to analyze the number of regular vs. altered mitochondria, followed by a Bonferroni post-hoc test. A *p* value of 0.05 or less was considered statistically significant (*). For immunofluorescence staining a minimum of 100 cells/sample per experiment, were analyzed for fluorescence intensity levels. A *p* value of 0.05 or less was considered statistically significant (*), while (**) indicate *p* values equal or lower than 0.01.
